# Narrow Versus Wide Diameter Portacaval H-Graft Shunts

**DOI:** 10.1155/1995/24527

**Published:** 1995

**Authors:** J. Michael Henderson

**Affiliations:** Department ofGeneral Surgery The Cleveland Clinic Foundation 9500 Euclid Avenue Cleveland Ohio 44195 USA


and therapy. Review of the literature from 1976–1981. *World J. Surg.*, **8**, 2.
9. Tanaka, H., Iwai, A., Sugimoto, H. (1991) Intrahepatic arterioportal fistula after blunt hepatic trauma: case reports. *J. Trauma.*, **31**, 143–146.
10. Noyes, L.D., Doyle, D.J., McSwain, N.E. (1988) Septic complications associated with the use of peritoneal drains in liver traumas. *J. Trauma.*, **28**, 337.
11. Gillmore, D., McSwain, N. E. Jr., Browder, I.W., (1987) Hepatic trauma: To drain or not to drain? *J. Trauma.*, **27**, 898.
12. Cogbill, T.H., Moore, E.E., Jurkovitch, G.J. *et al.* (1988) A multi-center experience with 1, 335 liver injuries. *J. Trauma.*, **28**, 14–33.
13. Pachter, H.L., Spencer, F.C., Hofstetter, S.R., Liang, H.G., Coppa, G.F. (1992) Significant trends in the treatment of hepatic trauma: Experience with 411 injuries. *Ann. Surg.*, **215**, 492–502.
14. Moore, E.E., Moore, J.B., Van Duzzer- Moore, S. *et al.* (1980) Mandatory laparotomy for gunshot wounds penetrating the abdomen. *Am. J. Surg.*, **140**, 847.

H. Leon Pachter, M D
Professor of Surgery
New York University School Of Medicine
Director, Trauma-Shock Unit
Bellevue Hospital, New York City, N.Y.

### NARROW VERSUS WIDE DIAMETER PORTACAVAL H-GRAFT SHUNTS

### ABSTRACT

*Sarfeh James, I. and Rypins, Eric, B. (1994) Partial versus total portacaval shunt in alcoholic cirrhosis. Annals of Surgery; 219, 353–361.*

***Objective.*** **Results of the first prospective randomized clinical trial comparing partial and total portacaval shunt for variceal hemorrhage are reported.**
***Summary Background Data.*** **Total portacaval shunts produce subnormal portal pressures, completely diverting hepatic portal flow. Partial shunts maintain higher pressures and preserve hepatopedal flow. No randomized trials of these two approaches have been performed.**
***Methods.*** **Alcoholic patients with cirrhosis (n = 30) and variceal hemorrhage treated at one institution were randomized to receive partial (8-mm diameter portacaval H grafts with collateral ablation, n = 14) or total shunts (16-mm diameter grafts, n = 16). Portography was performed after operation and then yearly. Investigators blinded to shunt type assessed encephalopathy; hospitalizations were reviewed.**
***Results.*** **Child's class, age and operative urgency were similar for the two groups. Two patients (with total shunts) died within 30 days. Hepatopedal flow was maintained in 13 partial and 0 total shunt patients ($p < 0.0001$). Shunt gradients were $16 \pm 5$ compared with $6 \pm 3$ cm saline after partial and total shunts ($p < 0.0001$). There were no shunt thromboses or variceal hemorrhages. Encephalopathy-free survival was significantly greater after partial shunts ($p = 0.013$; life table analysis). Five total compared with zero partial shunt patients required hospitalization for coma ($p = 0.02$). Long-term survival was not different for the two groups of patients.**
***Conclusions.*** **Partial shunts control variceal hemorrhages while maintaining hepatopedal flow and elevated portal pressures. By minimizing encephalopathy rates, partial shunts provide improved quality of survival compared with total shunts.**

KEY WORDS: Portacaval shunt sarfeh shunt.

### PAPER DISCUSSION

Partial portal decompression - does it really work? This carefully conducted prospective randomized controlled trial comparing partial to total portal decompression presents the most persuasive data yet available that partial decompression offers a significant advantage compared to total portal systemic shunt. This study needed to be done, and the authors are to be commended for a well designed and carefully executed study[1]. As two of the major advocates of partial shunting, Sarfeh and Rypins recognized the importance of appropriate study design and elimination of bias, as far as possible, in setting up this trial, I believe they have achieved this.

*Control of bleeding.* The primary goal of any treatment for variceal bleeding must be to control the bleeding. Data in this study supports that previously published [2,3] that a partial shunt is equally good as a total shunt in control of bleeding. There was no thrombosis or stenosis in either group, a factor which is particularly pertinent in the era of transjugular intrahepatic portalsystemic shunts (TIPS). Stenosis and/or thrombosis is the major problem with TIPS [4] and may limit its applicability as bleeding recurs in these patients.

*Encephalopathy.* The advantage clearly shown for partial shunting is a significantly ($p < .013$) lower rate of encephalopathy compared to patients receiving total portal systemic shunts. Let us examine this further.

Who were the patients? All were male, all had alcoholic cirrhosis, 90% were child's class A and B, and none had chronic or spontaneous encephalopathy prior to their shunts. The two groups were well matched, with the type of shunt being the only variable. Did they all stop drinking? The authors acknowledge that they could not define this accurately. This variable may have had some influence, but it is reasonable to assume was not significantly different between groups.

How were patients managed? Diet was not restricted in either group, nor were specific medications used prophylactically to reduce the risk of encephalopathy. For the purpose of this study this was an appropriate design when comparison between the two groups was the goal. No data are given as to whether protein intake was indeed equivalent between the two groups: unrestricted diet may not equate to equivalency! Other potential precipitants of encephalopathy, such as gastrointestinal bleeding, and infection do not appear different between groups. The greater diuretic requirement in the partial shunt group was a possible adverse factor in that group, but did not translate into a higher rate of encephalopathy.

How was encephalopathy assessed? This is always one of the most difficult design factors in a study such as this. The authors chose to use two endpoints, i) a "functional" encephalopathy measure, and ii) the need for rehospitalization. The differences between the two shunt groups is very clear by any index used, - and the 57% incidence of moderate to severe encephalopathy in the total shunt group compared to 14% in the partial shunt group is the most compelling data in this study. Use of other tests of encephalopathy such as EEG or psychometric evaluation might be sought by some purists, but are unlikely to have altered this finding.

In parallel with prior data from this group [2], the low encephalopathy rate in the partial shunt group is associated with good maintenance of portal perfusion to the liver. Unfortunately, less than 50% of patients had hemodynamic studies at late follow up, which weakens the conclusions for the hemodynamic/encephalopathy correlation at that time.

*Other end points.* Ascites was significantly higher in the partial shunt group. This is not surprising as portal pressure was not reduced as much in this group. This finding does raise the question as to why a greater portal pressure reduction is required to control ascites than bleeding in portal hypertension?

Survival was not significantly different between the two groups at 20 months. Longer term follow up in this study is important to document if the markedly lower encephalopathy of the partial shunt group will ultimately be associated with better survival.

Where does partial shunting fit in the total scheme of management?

In the 1990s, most would agree there is no single best way to manage all patients with variceal bleeding. The range of options for such patients now goes from pharmacologic portal pressure reduction, to sclerotherapy, radiologic shunts, total, partial or selective operative decompression, devascularization, all the way to liver transplantation. Better understanding of the underlying diseases and their natural history, of therapies and their natural history, and an ability to match patients to their appropriate therapy is the key in the 1990's. The data in this paper provide useful information in defining a role for partial systemic shunt.

### *References*

1. Sarfeh, I. J., Rypins, E.B. (1994) Partial versus total portacaval shunt in alcoholic cirrhosis. *Ann. Surg.*, **219**, 353–361.
2. Sarfeh, I. J., Rypins, E.B., Manson, G. R. (1986) A systematic appraisal of portacaval H-graft diameters: Clinical and hemodynamic perspectives. *Ann. Surg.*, **204**, 356–363.
3. Rosemurgy, A.S., McAllister, E.W., Kearney, R.E. (1991) Prospective study of a prosthetic H-graft portacaval shunt. *Am. J. Surg.*, **161**, 159–164.
4. LaBerge, J. M., Ferrell, L.D., Ring, E. J. *et al.* (1993) Histopathologic study of stenotic and occluded transjugular intrahepatic portosystemic shunts. *J. VIR.*, **4**, 779–786.

J Michael Henderson FRCS, M D
Chairman

Department of General Surgery
The Cleveland Clinic Foundation
9500 Euclid Avenue
CLEVELAND, OHIO 44195
United States of America

